# Estimating soil water retention for wide ranges of pressure head and bulk density based on a fractional bulk density concept

**DOI:** 10.1038/s41598-020-73890-8

**Published:** 2020-10-07

**Authors:** Huihui Sun, Jaehoon Lee, Xijuan Chen, Jie Zhuang

**Affiliations:** 1 Department of Biosystems Engineering and Soil Science, The University of Tennessee, Knoxville, TN 37996 USA; 2 Key Laboratory of Pollution Ecology and Environmental Engineering, Institute of Applied Ecology, Chinese Academy of Sciences, Shenyang, 110016 China; 3 Center for Environmental Biotechnology, The University of Tennessee, Knoxville, TN 37996 USA

**Keywords:** Ecology, Environmental sciences, Hydrology

## Abstract

Soil water retention determines plant water availability and contaminant transport processes in the subsurface environment. However, it is usually difficult to measure soil water retention characteristics. In this study, an analytical model based on a fractional bulk density (FBD) concept was presented for estimating soil water retention curves. The concept allows partitioning of soil pore space according to the relative contribution of certain size fractions of particles to the change in total pore space. The input parameters of the model are particle size distribution (PSD), bulk density, and residual water content at water pressure head of 15,000 cm. The model was tested on 30 sets of water retention data obtained from various types of soils that cover wide ranges of soil texture from clay to sand and soil bulk density from 0.33 g/cm^3^ to 1.65 g/cm^3^. Results showed that the FBD model was effective for all soil textures and bulk densities. The estimation was more sensitive to the changes in soil bulk density and residual water content than PSD parameters. The proposed model provides an easy way to evaluate the impacts of soil bulk density on water conservation in soils that are manipulated by mechanical operation.

## Introduction

Modeling of water flow and chemical movement in unsaturated soils has been emphasized by soil scientists and hydrologists for different purposes, such as evaluations of root water uptake, soil erosion, and groundwater pollution risk. However, high variability and complexity of soil texture in natural field make direct measurements of soil hydraulic properties costly and time-consuming. It is desirable to utilize readily available information, such as soil texture and bulk density, to estimate soil hydraulic properties^1–3^. This kind of approach benefits the development of computationally efficient methods for evaluating soil hydraulic heterogeneity in watershed or agricultural field while ensuring the economic feasibility of field investigation efforts within acceptable accuracy. To date, many modeling efforts have been made to relate soil texture (expressed as particle size distribution), soil structural properties, bulk density, and/or organic matter content to soil water retention^4–7^. Soil water retention was estimated using multiple regression, neural network analyses, and other methods^8–14^. However, the applicability and accuracy of the models are more or less unsatisfactory. Several prediction models were derived on global soil hydraulic datasets, such as applying the Miller-Miller scaling approach to the soil dataset of SoilGrids1km to provide a global consistent soil hydraulic parameterization^15^, but some of them possess a high correlation to particular soil types and thereby may not be suitable for other soils^16–18^.

An important advancement in using soil particle size distribution to derive a soil water retention characteristic was the development of a physical empirical model by Arya and Paris^19,20^, Later, Haverkamp and Parlange^21^ proposed a similar model by combining physical hypotheses with empirical representations and tested the model on sandy soil. Tyler and Wheatcraft^22^ interpreted the empirical scaling parameter *α* in the Arya and Paris model as being equivalent to the fractal dimension of a tortuous fractal pore system. However, Arya et al.^20^ argued that the fractal scaling was limited in estimating water retention characteristics in the complex soil matrix. In the optimized model of Arya et al.^20^, three methods were proposed for calculating the scaling parameter *α*, but the calculation still involved empirical component to some extent, making the model sometimes relatively difficult for broad application. The physical basis of the model of Arya and Paris^19^ or Arya et al.^20^ is weakened by the assumption that the void ratio of bulk sample is equivalent to the void ratio of individual particle size class.

To improve the mathematical description of physical relations between soil particles and soil pores, we assume that different fractions of soil particles may make different contributions to the total porosity or volumetric water content in the bulk soils and that soil pore volume and associated bulk density are specific for particle size fractions. This line of thinking might help derive a better physical model for mathematical estimation of soil water characteristics. Therefore, the objective of this work was to apply a fractional bulk density (FBD) concept to the development of a soil water retention model that is effective for all soil textures and a wide range of soil bulk density.

## Results

### Estimation accuracy

**Figure 1:**
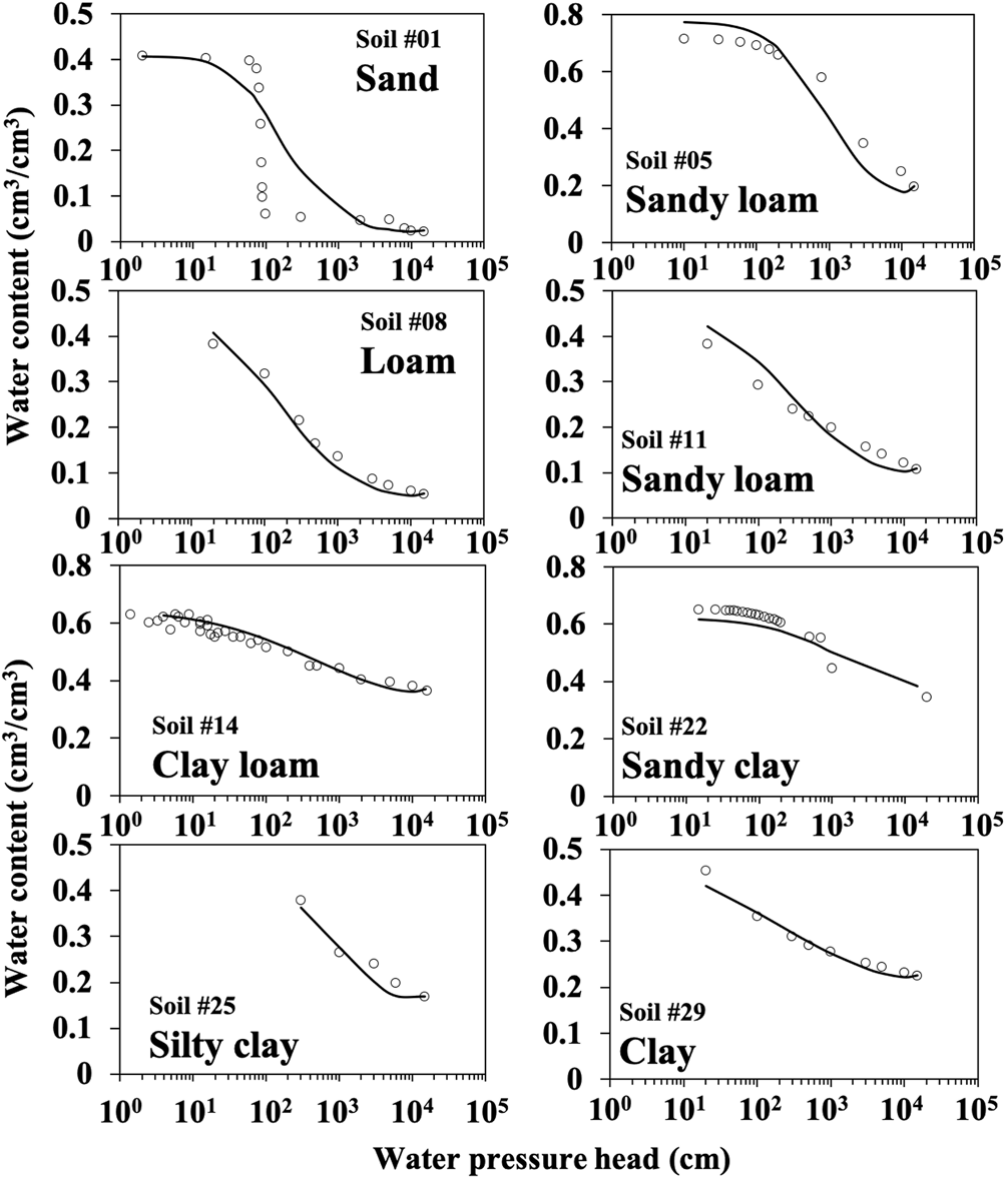
Water retention characteristics measured (circle) and estimated (line) using the fractional bulk density (FBD) model for eight different soil textures.

Model estimation of water retention characteristics for some soils is presented in Fig. 1. The results indicate that the new procedure was in good agreement with the measured data for most of the soil textures except for sand in the range of water pressure head from 15 cm to 15,000 cm, which covers the entire range of available water content. Table 1 shows comparisons of the coefficient of determination (*R*^2^), root mean square error (*RMSE*), and *t* value of Student’s *t* distribution between the FBD model and the curve fitting using the Campbell model^23^, which was extended from the similar media concept^24^. The Campbell model is expressed as

1 $${\theta} = \left( {\frac{{\psi}_{e}}{\psi}} \right)^{\frac{1}{q}} {\theta}_{s}$$

**Table 1:** Statistical comparison of soil water contents estimated by the fractional bulk density (FBD) model and fitted by the Campbell model^23^.

Soil No.	*t*		*R*^2^		*RMSE*		*n*
	FBD model	Campbell model	FBD model	Campbell model	FBD model	Campbell model	
01	1.600	− 0.508	0.662	0.507	0.097	0.117	16
02	− 1.316	− 0.288	0.939	0.888	0.039	0.040	9
03	− 1.924	− 0.213	0.982	0.958	0.011	0.014	5
04	− 0.123	− 0.308	0.968	0.932	0.012	0.015	5
05	− 0.191	0.115	0.962	0.900	0.059	0.060	10
06	− 1.374	− 0.331	0.951	0.922	0.068	0.039	5
07	− 1.948	− 0.123	0.908	0.922	0.027	0.018	5
08	− 2.411	0.125	0.985	0.942	0.020	0.031	9
09	0.532	− 0.270	0.954	0.923	0.017	0.024	5
10	1.345	0.179	0.980	0.998	0.042	0.003	9
11	0.144	0.164	0.977	0.995	0.028	0.007	9
12	0.266	0.070	0.995	0.964	0.020	0.020	9
13	10.840	− 0.819	0.938	0.899	0.029	0.010	6
14	3.572	3.885	0.951	0.956	0.023	0.024	28
15	− 0.883	− 0.128	0.932	0.980	0.028	0.013	10
16	− 0.209	− 0.163	0.876	0.877	0.062	0.057	16
17	− 0.794	− 0.056	0.908	0.958	0.027	0.014	5
18	− 0.441	− 0.227	0.957	0.967	0.019	0.014	10
19	− 0.256	− 0.065	0.891	0.936	0.027	0.017	5
20	1.153	− 0.253	0.892	0.920	0.032	0.023	10
21	1.932	− 0.156	0.908	0.928	0.030	0.020	10
22	− 5.494	− 0.218	0.945	0.881	0.035	0.024	20
23	− 3.341	− 0.067	0.922	0.966	0.056	0.015	5
24	− 1.421	− 0.061	0.939	0.971	0.023	0.012	5
25	− 1.558	− 0.077	0.938	0.970	0.023	0.013	5
26	1.707	− 0.075	0.953	0.969	0.022	0.010	10
27	− 0.108	− 0.052	0.916	0.963	0.020	0.013	5
28	− 0.346	− 0.113	0.958	0.956	0.014	0.014	9
29	− 0.026	0.016	0.968	0.969	0.014	0.012	9
30	− 1.823	− 0.032	0.952	0.965	0.021	0.013	5
Mean	0.375	− 0.009	0.934	0.931	0.032	0.024	

*t* is the value of Student’s t distribution, and the critical values of *t*_0.05_ for 04, 08, 09, 10, 15, 18 and 30 degrees of freedom are 2.776, 2.306, 2.262, 2.228, 2.131, 2.101 and 2.042, respectively; *R*^2^ is determination coefficient; *RMSE* is root mean square errors (cm^3^/cm^3^); *n* is the number of measured pairs of water content and pressure head.

where *ψ*_*e*_ is air-entry water potential, *θ*_*s*_ is saturated volumetric water content, and *q* can be obtained using

2 $${q} = \left[ {\sum {M}_{i} \left(\ln {D}_{gi}\right)^{2} - \left(\sum {M}_{{{i}}} \ln {D}_{gi} \right)^{2} } \right]^{0.5}$$

In the equation, *D*_*gi*_ is the diameter of the *i*th particle-size fractions, and *M*_*i*_ is the cumulative mass percentage of the ≤ *D*_*gi*_ particles.

**Figure 2:**
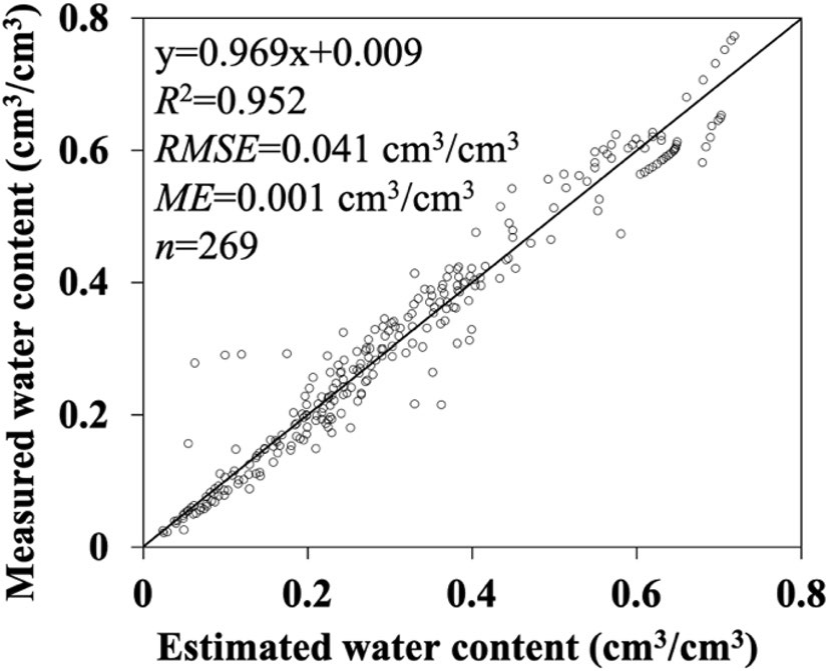
Comparison of measured and estimated volumetric water content using the fractional bulk density (FBD) model for 30 soils with ranges of soil texture from clay to sand and bulk density from 0.33 to 1.65 g/cm^3^. The circle represents measured values, and the line denotes a 1:1 agreement.

*RMSE* values were computed from soil water contents measured and estimated as described in the section of methods. Table 1 shows that the mean value of *RMSE* of the FBD model was 0.032 cm^3^/cm^3^ while that of the Campbell model was 0.024 cm^3^/cm^3^. This result was acceptable because the Campbell model used the measured data to fit *ψ*_*e*_. The *R*^2^ values also supported the acceptability of the FBD model compared to the Campbell model. According to the *t* values, the FBD model results had no significant difference and systematic bias from the measurements for 25 out of the 30 soils. Figure 2 shows an overall comparison between the water contents measured and estimated by the FBD model for the 30 soils. The values coalesced to the 1:1 line with the *RMSE* being 0.041 cm^3^/cm^3^. This *RMSE* value was larger than the average in Table 1. The discrepancy was due that different methods were used for averaging the *RMSE* values for individual soils and all soils. Mayr and Jarvis^25^ presented pedotransfer functions to estimate soil water retention parameters of the Brooks–Corey model. The resulting mean *RMSE* value was 0.043 cm^3^/cm^3^ for the dependent dataset and 0.048 cm^3^/cm^3^ for the independent dataset. Tomasella et al.^26^ derived a pedotransfer function to predict the water retention parameters of the van Genuchten equation. The mean *RMSE* values ranged from 0.038 cm^3^/cm^3^ to 0.058 cm^3^/cm^3^. Our model compared favorably with these pedotransfer functions in terms of mean *RMSE* values. It could thus be concluded that the FBD model behaved overall well, except for Acolian sandy soil (Soil #01). For sandy soil, the relatively poor capture of the rapid change of water content was attributed to the limitation of applicability of capillary law (i.e., Young–Laplace equation) to sandy media and existence of macropores that might reduce the pore continuity^8^. The continuity of soil pores was the dominant factor that affected the performance of our proposed model.

The FBD model also had relatively larger estimation errors for soils originated from ash parent materials (e.g., Soil #05, 14, 16, and 22) than for other soils (Table 1). This was due likely to the oversimplification of soil particle size distribution as a sigmoid curve, whereas the particle arrangement of soils developed from ash parent materials was actually very complex (i.e., non-sigmoid). The less accurate prediction for sandy soils relative to the other soil textures suggested that the sigmoid-shape assumption of particle size distribution might be arbitrary, despite it was well applied to the particle systems of other soil textures. We infer that the sigmoid-type distribution was more applicable to the soils with a broader range of particle sizes, which demonstrated a lognormal distribution of particle fractions^27,28^. Soil aggregates with hierarchical pore structure have dual-porosity system. Dual-porosity assumes that porous medium consists of two interacting regions, one associated with the macropore or fracture system and the other comprising micropores inside soil material. Bimodal pore size distributions are frequently observed in dual-porosity soil^29^. The water retention estimated with the FBD model for a wide range of water pressure head (15–15,000 cm) should thus be a sum of the effects of macropores and micropores^30^. The sigmoid-type distribution should be more suitable for hierarchical soil aggregates than for less structured soils, such as sandy soil whose pore system was simply dominated by primary particles. Therefore, the FBD model might not perform very well against the soils if their particle sizes have a narrow range.

### Sensitivity analysis of model parameters

**Figure 3:**
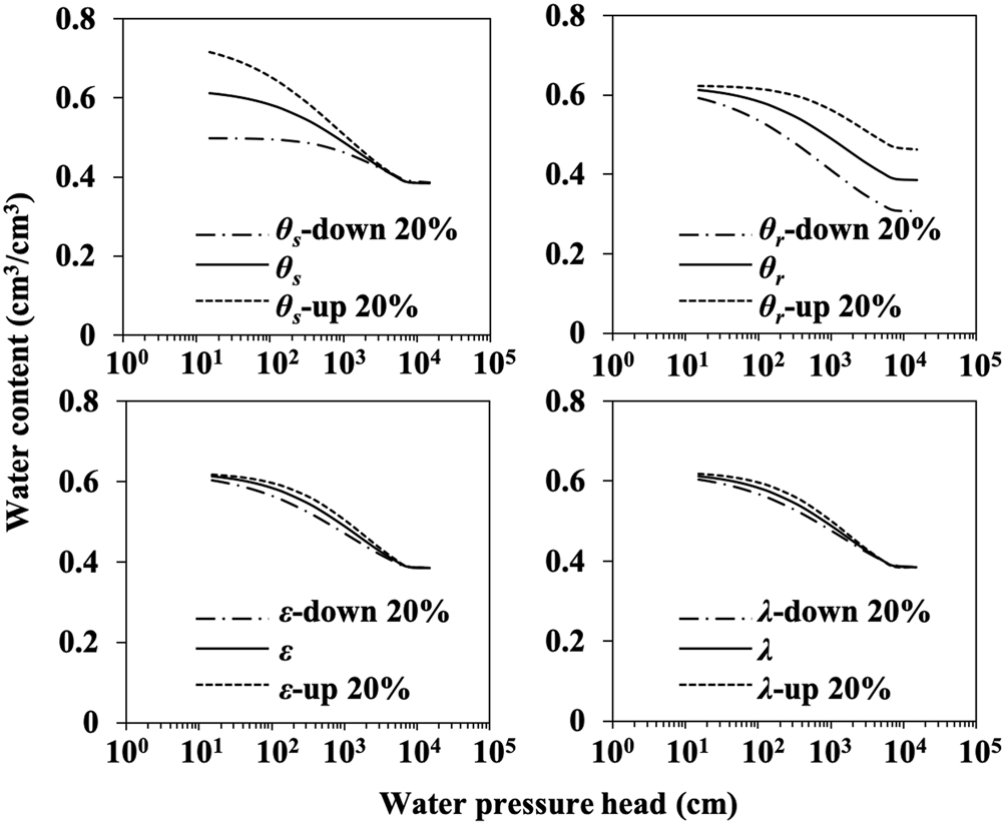
A sensitivity analysis on the parameters of the fractional bulk density (FBD) model (Eq. 20). The analysis was based on a sandy clay soil (Andisols, Soil #22 in Table 2). *θ*_*s*_, *θ*_*r*_, *ε*, and *λ* refer to volumetric saturated water content, volumetric residual water content at a pressure head of 15,000 cm water, particle size distribution index, and rate coefficient in Eq. (15) for particle size distribution, respectively.

We performed a sensitivity analysis to identify input parameters that most strongly affected the model behavior and to determine the required precision of the key parameters. The parameters included in the sensitivity test were saturated water content (*θ*_*s*_), residual water content (*θ*_*r*_), rate coefficient (*λ*) of Logistic-type model for particle size distribution, and particle size distribution index (*ε*). The value of each parameter was assumed to increase or decrease by 20% of its actual value since its measurement error could be up to 20% according to our experience in field survey. By taking Soil #22 as an example, the test was implemented to monitor the change in the estimated soil water content caused by changing the value of one parameter at a time while others remained constant. The sensitivity analysis not only showed the influence patterns of the parameters on the model behavior but also ranked the parameters in terms of the magnitude of influences. Figure 3 shows that *θ*_*s*_ and *θ*_*r*_ had similarly large impacts on the model estimation. In comparison, *λ* and *ε* played less roles in defining the model performance, but their accuracy was still very important for the estimation accuracy. The sensitivity analysis provided insights into the behavior of the FBD model (Eq. 20) and supported the notion that parameter values may have physical meanings no matter in whatever ways the related parameters are structured into a model.

## Discussion

Particle size distribution forms a common descriptor of natural soils. It has been used routinely as one of the inputs to estimate some of soil physical properties, for example, water retention characteristic^31–33^, bulk density^34^, and hydraulic conductivity^35–37^. In this study, two parameters, rate coefficient (*λ* in Eq. 15) of the Logistic-type model for particle size distribution and particle size distribution index (*ε* in Eq. 13), were employed to translate particle size distribution to soil water retention characteristic. However, two parameterization issues should be mentioned for broadening model applicability. One is the estimation of *λ* in the case that the upper size limit of the particle size distribution is 1,000 μm for some soils while it is 2,000 μm for other soils. In order to perform a consistent comparison among all soils, the particle size distribution with the upper limit of 2,000 μm was normalized to that with the upper limit of 1,000 μm using a normalization formula,

3 $${{M}}^{\prime}_{{{i}}} = \frac{{{{100M}}_{{{i}}} }}{{{{M}}_{{1,000}} }}$$

where *M*_*i*_ and *M*_*i*_*’* are measured and normalized percentage content of particles with sizes smaller than or equal to the *i*^th^ particle size, respectively. *M*_1,000_ denotes the mass percentage of particles with a diameter smaller than or equal to 1,000 μm. The other issue is pertinent to the calculation of *ε*. It involved three particle sizes (*D*_10_, *D*_40_, and *D*_60_) below which the mass percentage of particles is 10%, 40%, and 60%, respectively. It is easy to identify *D*_60_ but sometimes relatively difficult to find *D*_10_ and *D*_40_. In some soils, the mass of particles with sizes smaller than or equal to the measured lower limit size (e.g., 1 μm or 2 μm) was larger than 10%. In this case, an exponential equation, which was obtained by fitting the relation between the cumulative mass percentage and the corresponding particle sizes, was used to extrapolate for estimating *D*_10_. To minimize the deviations arising from the extrapolation, we used 50 μm as the upper size limit of the particle size distribution.

There is no doubt that particle assembling and resulting pore characteristics play important roles in regulating physical, chemical, and biological functions of soils at various scales. The FBD model was generally based on the assumption that the sizes of soil particles and the density of their packing are the primary determinants of the pore size and pore volume. This, however, may not be the case under some conditions. Aggregation of primary particles into secondary and tertiary particles, root channels, and microcracks would account for a fraction of the pore volume with pore sizes not determined by the size distribution of primary particles. The abundance of such pores considerably determines the extent of deviation of prediction. Therefore, it is important to incorporate information of soil structure into soil hydraulic modeling if possible^38^. Soil structure is a non-negligible factor for accurate estimation of soil hydraulic properties using pedotransfer functions^39,40^. But this work is difficult to initiate because soil structure information (e.g., soil aggregate size distribution) is mostly unavailable compared to soil basic properties (e.g., particle size distribution, organic matter content, and bulk density). Insufficiency of identification of soil structure indices precludes the inclusion of soil structure characteristics into soil water retention modeling. Relevant efforts have been made in some large-scale models that consider soil structure. For instance, Fatichi et al.^41^ proposed to assess the impact of soil structure on global climate using an Ocean-Land–Atmosphere Model (OLAM). Although the model in this study does not explicitly include a structural component, in the FDB model we assume that soil bulk density could indirectly bring the influence of soil structure into the estimation of soil water retention.

Soil water retention characteristics were estimated using the FBD model from particle size distribution, bulk density, and measured residual water content. The starting point was the similarity of curve shapes between cumulative particle size distribution and soil water retention characteristics. Similarly, Arya and Paris^19^ and Haverkamp and Parlange^21^ used a simple equation to derive a set of soil water content according to the mass fraction of soil particles, and then a series of expressions were employed to regulate soil water pressure head to pair with measured soil water content. The FBD model adopted an opposite approach. A set of water pressure head from 15 cm to 15,000 cm were derived using a simple expression as Eq. (19), and then soil water contents were estimated with Eq. (8) to match the derived water pressure head. Eventually, an analytical model (Eq. 20) was obtained. In the FBD model, the water retention function included a residual water content in relation to the maximum water pressure head (15,000 cm) and the parameter (*b*) of soil pore size distribution. Similarly, the residual water content was considered in the van Genuchten model^42^ or Brooks and Corey model^43^. However, Campbell^23^ described soil water retention curve by assuming there was no residual water content. An advantage of the Campbell equation is its excellent fitting capability. Thus, we evaluated the performance of the FBD model by comparing it to the Campbell model in this study.

The selection of a Logistic-type equation for the model formulation was mainly due to the consideration that particle size distribution and pore size distribution in most soils were approximately lognormal^27,44–46^. The logistic growth equation generated a curve that tended towards an exponential form at low values and a power form at high values, with a power index smaller than 1. This characteristic implicitly included the consideration that the drainage of water in small pores at large suction was usually expected to be more impaired than the release of water from large pores at small suctions^47,48^.

## Conclusions

An analytical model, which is based on a fractional bulk density concept, was presented for estimating soil water retention for the entire range of water pressure head that determines water availability. The proposed model was tested using 30 sets of soil water retention data measured for various textures of soils that had a wide range of soil bulk density from 0.33 g/cm^3^ to 1.65 g/cm^3^. Results showed that the proposed model could convert readily available soil physical properties into soil retention curves in very good agreement with the measurements, and the model was applicable to soils with limited data of soil particle size distribution at small loss of estimation accuracy in the middle portion of water retention curves of sandy soils. Sensitivity analyses revealed that saturated and residual water contents were two parameters of high sensitivity for accurate estimation of the water retention curves. The agreement between the estimated and measured results supported the concept underlying the FBD model. The modeling followed a process of conceptual partitioning of pore space according to the relative contribution of certain sizes of particles to the change in pore space. In addition, the model assumed a sigmoid curve of water retention characteristic for most soils. However, these assumptions need further verification by considering the physical reality of soils and potential improvements and extensions. Compared to subsurface soils, larger deviations should be expected for surface soil materials where aggregation, cracking, and root effects may be pronounced. Further tests of the model application to other soils (e.g., Vertisols, Aridisols, and salt affected soils) and evaluation of the effects of water hysteresis, soil aggregation, and swelling-shrinkage behaviors might reveal the weaknesses of the FBD model and help identify additional variables needed for model improvement.

## Material and methods

### Fractional bulk density concept

**Figure 4:**
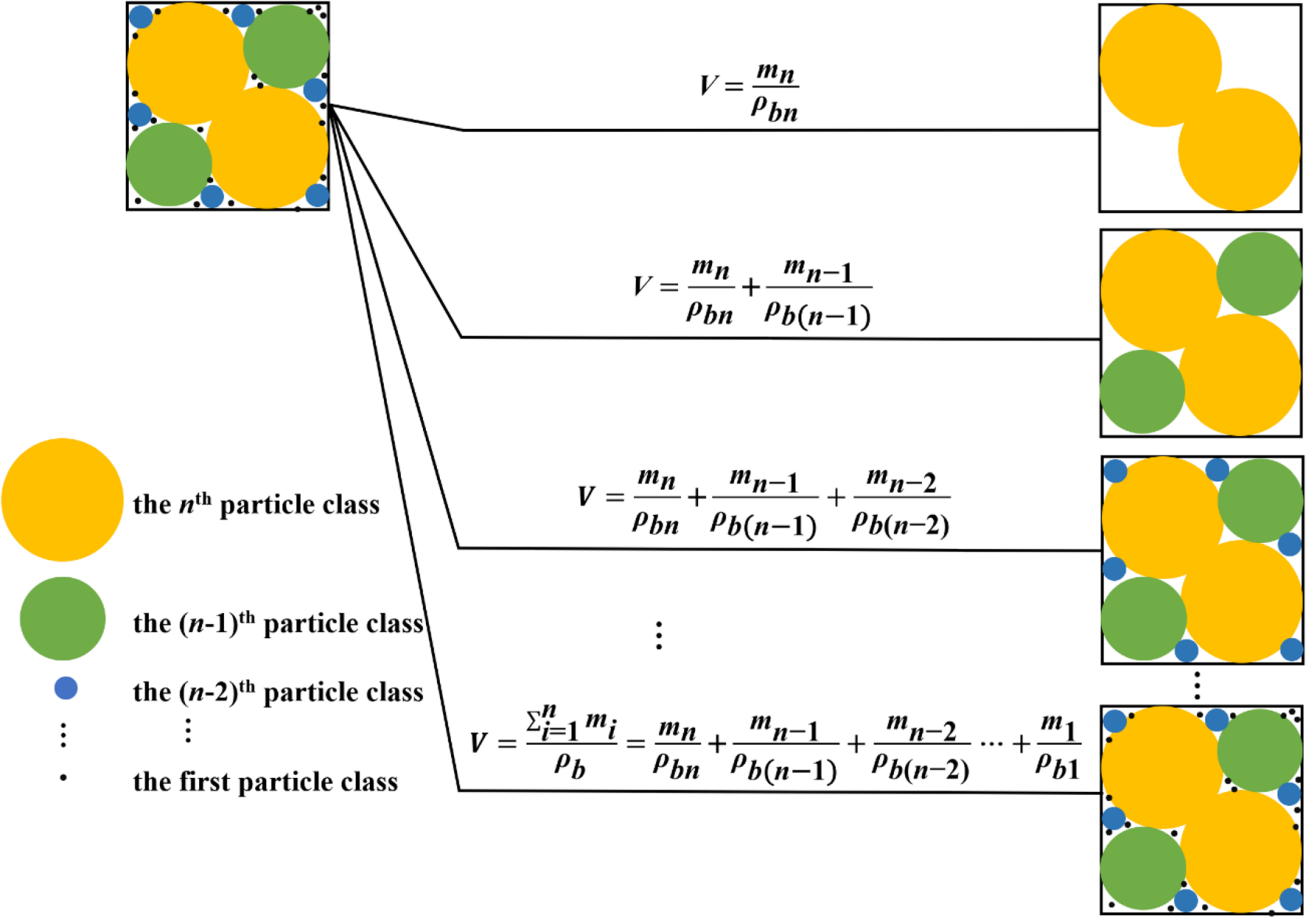
Diagrammatic representation of the fractional bulk density (FBD) model.* V* and *ρ*_*b*_ are the volume of bulk soil and the bulk density of whole soil, respectively. *m*_*i,*_ and *ρ*_*bi*_ refer to the solid mass and equivalent bulk density associated with the *i*th particle-size fractions, respectively.

The first assumption is that soil particles with different sizes contribute to different porosities and water holding capacities in bulk soil. Based on a non-similar media concept (NSMC) defined by Miyazaki^49^, soil bulk density (*ρ*_*b*_) is defined as

4 $$\rho _{{{b}}} = \frac{{{M}}}{{{V}}} = \tau \rho _{{{s}}} \left( {\frac{{{S}}}{{{{S}} + {{d}}}}} \right)^{3}$$

where *M* is the mass of a given soil, *V* is the volume of bulk soil, *ρ*_*s*_ is soil particle density, and *S* and *d* are characteristic lengths of solid phase and pore space, respectively. The parameter *τ* is a shape factor of the solid phase, defined as the ratio of the substantial volume of solid phase to the volume *S*^3^. The value of *τ* is 1.0 for a cube and π/6 for a sphere. As pointed out by Miyazaki^49^, these characteristic lengths are not directly measurable but are representative lengths in the sense of the characteristic length in a similar media concept (SMC). Following the approach of NSMC represented by Eq. (4), we conceptually defined the volume of bulk soil as

5 $$V = \frac{{\mathop \sum \nolimits_{{{i } = { 1}}}^{{{n}}} {{m}}_{{{i}}} }}{{{\rho }_{{{b}}} }} = \frac{{{{m}}_{{1}} }}{{{\rho }_{{{{b1}}}} }}{ + }\frac{{{{m}}_{{2}} }}{{{\rho }_{{{{b2}}}} }}{ + } \cdot{\mkern -4mu}\cdot{\mkern -4mu}\cdot \frac{{{{m}}_{{{n}}} }}{{{\rho }_{{{{bn}}}} }}$$

where *m*_*i*_ and *ρ*_*bi*_ are the solid mass and equivalent bulk density of the *i*th size fraction of soil particles, respectively. In this study, diameters of the first particle fraction and the last one were assumed to be 1 µm and 1000 µm, respectively^8^. This equation suggests that different particle size fractions are associated with different equivalent bulk densities due to different contributions of particle arrangement to soil pore space. As a result, the particles with the same size fraction could have different equivalent bulk densities in soils with different textures or after the soil particles are rearranged (e.g., compaction). Figure 4 provides a diagrammatic representation of such fractional bulk density concept for the variation of soil pore volume with soil particle assemblage.

### Calculation of volumetric water content

For a specific soil, Eq. (5) means

6 $${{V}}_{{{{pi}}}} \left( { \le {{D}}_{{{i}}} } \right){{ } = { f}}\left( {{{D}}_{{{{gi}}}} {{, M}}_{{{i}}} } \right)$$

where *V*_*pi*_(≤ *D*_*i*_) denotes the volume of the pores with diameter ≤ *D*_*i*_ generated by soil particles with diametes ≤ *D*_gi_ in unit volume of soil. *M*_*i*_ is the cumulative mass percentage of the ≤ *D*_*gi*_ particles. Since the pore volume has the maximum value for a given bulk soil and the cumulative distribution of pore volume could be generally hypothesized as a sigmoid curve for most of the natural soils^44,45^, we formulated Eq. (6) using a lognormal Logistic equation,

7 $${{V}}_{{{{pi}}}} \left( { \le {{D}}_{{{{gi}}}} } \right) = \frac{{{{V}}_{{{{pmax}}}} }}{{1 + \kappa \left( {{{D}}_{{{{gi}}}} } \right)^{{{{b}}_{{{i}}} }} }}$$

where *V*_*pmax*_ is the maximum cumulative volume of pores pertinent to the particles smaller than or equal to the maximum diameter (*D*_*gmax*_) in unit volume of soil. In fact, here *V*_*pmax*_ is equal to the total porosity (*φ*_*T*_) of soil. *V*_*pi*_ (≤ *D*_*gi*_) is the volume of the pores produced by ≤ *D*_*gi*_ particles in unit volume of soil, and *b*_*i*_ is a varying parameter of increase in cumulative pore volume with an increment of *D*_*gi*_. By assuming a complete saturation of soil pore space, Eq. (7) changes into

8 $$\theta_{{{i}}} \left( { \le {{D}}_{{{{gi}}}} } \right) = \frac{{\theta_{{{s}}} }}{{1 + \kappa \left( {{{D}}_{{{{gi}}}} } \right)^{{{{b}}_{{{i}}} }} }}$$

where *θ*_*s*_ is saturated volumetric water content calculated with

9 $$\theta_{{{s}}} = \left\{ {\begin{array}{*{20}l} {0.9\varphi _{{{T}}} ,} \hfill & {~\rho _{{{b}}} < 1} \hfill \\ {\varphi _{{{T}}} ,} \hfill & {~~\rho _{{{b}}} \ge 1} \hfill \\ \end{array} } \right.$$

10 $$\varphi _{{{T}}} = \frac{{\rho _{{{s}}} - \rho _{{{b}}} }}{{\rho _{{{s}}} }}$$

In the above equations, *ρ*_*b*_is measured soil bulk density, and *ρ*_*s*_ is soil particle density (2.65 g/cm^3^). The empirical parameter *κ* in Eqs. (7) and (8) is defined as

11 $${{\kappa}} = \frac{{\theta_{{{s}}} - \theta_{{{r}}} }}{{\theta_{{{r}}} }}$$

where *θ*_*r*_ is measured residual water content. In this study, *θ*_*r*_ is set as the volumetric water content at water pressure head of 15,000 cm. The empirical parameter *b*_*i*_ is defined as

12 $${{b}}_{{{i}}} = \frac{{\epsilon }}{{3}}{\log}\left( {\frac{{{\theta }_{{{s}}} {{ - \omega }}_{{{i}}} {\theta }_{{{s}}} }}{{{{\kappa \omega }}_{{{i}}} {\theta }_{{{s}}} }}} \right)$$

with *ε*, a particle size distribution index, calculated with

13 $${\varepsilon }\; = \;\frac{{\left( {{{D}}_{{{40}}} } \right)^{{2}} }}{{{{D}}_{{{10}}} {{D}}_{{{60}}} }}$$

where *D*_10_, *D*_40_, and *D*_60_ represent the particle diameters below which the cumulative mass percentages of soil particles are 10%, 40%, and 60%, respectively.

The parameter *ω*_*i*_ is coefficient for soil particles of the *i*th size fraction, with a range of value between *θ*_*r*_/*θ*_*s*_ and 1.0. By incorporating soil physical properties, *ω*_*i*_ can be estimated with

14 $${\omega }_{{{i}}} = \frac{{{g}}}{{{{1 + \kappa }}\left( {{{lnD}}_{{{{gi}}}} } \right)^{{\lambda}} }}$$

where *g* is regulation coefficient (1.0–1.2). We set it to be 1.2 in this study. *λ* is the ratio coefficient of particle size distribution fitted using the lognormal Logistic model,

15 $$M_{i} = \frac{{M_{T} }}{{1 + \eta D_{{gi}} ^{\lambda } }}$$

where *M*_*T*_ represent the total mass percentage of all sizes of soil particles, and *η* is a fitting parameter. We set *M*_*T*_ = 101 in Eq. (15) for best fit of the particle size distribution. In this study, this continuous function was generated from the discrete data pairs of *D*_*gi*_ and *M*_*i*_ at cutting particle diameters of 1,000, 750, 500, 400, 350, 300, 250, 200, 150, 100, 50, 30, 15, 7.5, 5, 3, 2, and 1 μm. Considering the difference in the upper limits of particle sizes associated with existing datasets of *D*_*gi*_ and *M*_*i*_, the particle size distribution with the upper limit of 2,000 μm for the Acolian sandy soil and volcanic ash soils in Table 2 was normalized to the case with the upper limit of 1,000 μm using Eq. (3).

**Table 2:** Physical properties of soils used in the study. *ρ*_*b*_ is bulk density (g/cm^3^); *θ*_*r*_ is residual water content (cm^3^/cm^3^) at 15,000 cm water pressure head; ε is particle size distribution index.

No	Soil	USDA soil taxonomy	Texture	Particle percentage		*ρ*_*b*_	*θ*_*r*_	*ε*	Source
				< 2 μm	< 20 μm				
01	Acolian sandy soil	Entisols	Sand	0.11	0.53	1.65	0.024	1.37	^53^
02	Meadow soil	Inceptisols	Sandy loam	6.04	35.20	1.38	0.039	1.38	^51^
03	Fluvo-aquic soil	Inceptisols	Sandy loam	9.51	38.01	1.33	0.055	1.82	^52^
04	Fluvo-aquic soil	Inceptisols	Sandy loam	10.20	33.20	1.27	0.051	1.87	^52^
05	Volcanic ash soil	Andisols	Sandy loam	10.22	35.00	0.33	0.199	3.09	^55^
06	Fluvo-aquic soil	Inceptisols	Sandy loam	13.55	45.60	1.27	0.062	1.75	^52^
07	Fluvo-aquic soil	Inceptisols	Loam	10.76	42.40	1.32	0.088	1.65	^52^
08	Meadow soil	Inceptisols	Loam	13.27	44.37	1.28	0.054	1.74	^51^
09	Fluvo-aquic soil	Inceptisols	Loam	13.40	47.88	1.32	0.059	2.48	^52^
10	Purplish soil	Inceptisols	Loam	16.32	48.04	1.30	0.092	1.58	^51^
11	Yellow earth	Inceptisols	Silt clay loam	27.35	73.87	1.29	0.108	1.61	^51^
12	Meadow soil	Inceptisols	Clay loam	22.09	47.32	1.29	0.082	1.95	^51^
13	Fluvo-aquic soil	Inceptisols	Clay loam	28.86	58.39	1.28	0.159	2.21	^52^
14	Volcanic ash soil	Andisols	Clay loam	28.01	65.00	0.80	0.370	1.73	^53^
15	Chernozem soil	Mollisols	Sandy clay	30.14	48.56	1.24	0.148	4.57	^50^
16	Volcanic ash soil	Andisols	Sandy clay	34.56	45.60	0.70	0.263	1.57	^54^
17	Fluvo-aquic soil	Inceptisols	Sandy clay	36.22	76.05	1.29	0.185	2.15	^52^
18	Brown earth	Alfisols	Sandy clay	36.77	54.36	1.29	0.142	3.85	^50^
19	Fluvo-aquic soil	Inceptisols	Sandy clay	40.02	73.30	1.28	0.195	2.31	^52^
20	Cinnamon soil	Alfisols	Sandy clay	40.12	59.37	1.19	0.138	3.74	^50^
21	Black soil	Mollisols	Sandy clay	42.18	59.34	1.15	0.186	3.44	^50^
22	Volcanic ash soil	Andisols	Sandy clay	45.37	63.28	0.82	0.385	3.14	^53^
23	Fluvo-aquic soil	Inceptisols	Silty clay	34.20	73.98	1.31	0.148	2.10	^52^
24	Fluvo-aquic soil	Inceptisols	Silty clay	33.31	78.73	1.30	0.161	2.12	^52^
25	Fluvo-aquic soil	Inceptisols	Silty clay	33.56	79.44	1.35	0.169	2.17	^52^
26	Albic soil	Spodosols	Clay	52.76	77.60	1.16	0.230	1.66	^50^
27	Fluvo-aquic soil	Inceptisols	Clay	56.05	89.82	1.25	0.283	2.76	^52^
28	Red earth	Ultisols	Clay	58.88	79.26	1.22	0.195	1.03	^51^
29	Humid-thermo ferralitic	Oxisols	Clay	72.57	85.60	1.15	0.225	1.05	^51^
30	Fluvo-aquic soil	Inceptisols	Clay	68.81	98.02	1.08	0.303	2.04	^52^

The Soil water retention data of fluvo-aquic soil, red earth, humid-thermo ferralitic, purplish soil, meadow soil, and yellow earth were measured with pressure membrane apparatus^51,52^. The soil water retention data of black soil, chernozem soil, cinnamon soil, brown earth, and albic soil were obtained using the suction and pressure plate method^50^. The soil water retention data of volcanic ash soil and Acolian sandy soil were measured using the suction and pressure plate method^53–55^.

### Calculation of water pressure head

To estimate the capillary tube or pore diameter (*D*_*i*_ in µm), which was composed of particles with the size of *D*_*gi*_ (µm), Arya and Paris^19^ developed an expression

16 $${{D}}_{{{i}}} {{ } = { D}}_{{{{gi}}}} \left[ {\frac{{2}}{{3}}{{en}}_{{{i}}}^{{{{(1 - \alpha )}}}} } \right]^{{{0}{{.5}}}}$$

where *α* is the empirical scaling parameter varying between 1.35 and 1.40 in their original model^19^, but was thought to vary with soil particle size in the optimized model of Arya et al.^20^. In Tyler and Wheatcraft's model^22^ *α* is the fractal dimension of the pore. The parameter *e* is the void rate of entire soil and assumed unchanging with particle size. However, according to Eqs. (5) and (6), *e* in Eq. (16) should vary with particle size and be replaced by *e*_*i*_, which depends on soil particle sizes. *n*_*i*_ is the number of particles in the *i*th size fraction with a particle diameter (*D*_*gi*_ in μm), assuming that the particles are spherical and that the entire pore volume formed by assemblage of the particles in this class is represented by a single cylindrical pore. The equation for calculating *n*_*i*_ is given as^19^

17 $$n_{i} = \frac{{6m_{i} }}{{\rho_{s} \pi D_{gi}^{3} }} \times 10^{12}$$

where *m*_*i*_ is the mass of particles in the *i*th size fraction of particles. Assuming that soil water has a zero contact angle and a surface tension of 0.075 N/m at 25 °C, the minimum diameter of soil pore (*D*_*min*_) was taken to be 0.2 µm in this study, which is equivalent to the water pressure head of 15,000 cm according to Young–Laplace equation. We set this minimum pore size to correspond the minimum particle size (*D*_*gmin*_ = 1.0 µm). The FBD model might thus not apply well to porous media with pores smaller than 0.2 μm. As a result, Eq. (16) can be simplified into the following equation.

18 $${{D}}_{{{i}}} { = 0}{{.2D}}_{{{{gi}}}}$$

The equivalent capillary pressure (*ψ*_*i*_ in cm) corresponding to the *i*th particle size fraction can be calculated using

19 $$\psi_{{{i}}} = \frac{{{3000}}}{{{{D}}_{{{i}}} }} = \frac{{{15000}}}{{{{D}}_{{{{gi}}}} }}$$

In Eq. (19), the maximum water pressure head (*ψ*_*r*_ = 15,000 cm) corresponds to *θ*_*r*_ and *D*_*gmin*_ (1 μm). The minimum water pressure head (*ψ*_0_ = 15 cm) corresponds to *θ*_*s*_ and *D*_*gmax*_ (1,000 μm). These assumptions were arbitrary and might not be appropriate for some soil types. But these values were used in the study because they approximated the practical range of measurements well.

### The resulting model of soil water retention

Equations 8 and 19 formulate a FBD-based model for estimation of soil water retention curve. To simplify the computation, we incorporated the two equations into the following analytical form,

20 $${\theta }\; = \;\frac{{{\theta }_{{{s}}} }}{{{1 + }\left( {\frac{{{\theta }_{{{s}}} - {\theta }_{{{r}}} }}{{{\theta }_{{{r}}} }}} \right)\left( {\frac{{15,000}}{{\psi }}} \right)^{{{b}}} }}$$

with the parameter *b* obtained using

21 $${{b}}\; = \;\frac{{\epsilon }}{{3}}{\log}\left\{ {\frac{{{{(\theta }}_{{{s}}} - {\theta }_{{{r}}} {{)[ln(}}\frac{{{15,000}{{.1}}}}{{\psi }}{)]}^{{\lambda }} - {{(g}} - {{1)\theta }}_{{{r}}} }}{{{{g(\theta }}_{{{s}}} - {\theta }_{{{r}}} {)}}}} \right\}$$

In Eq. (21), a water pressure head of 15,000.1 cm is employed to consecutively predict the soil water content until the water pressure head of 15,000 cm.

### Soil dataset

Evaluation of the applicability of the proposed modeling procedure required datasets that included soil bulk density, residual water content, and soil particle size distribution covering three particle diameters (*D*_10_, *D*_40_, and *D*_60_) below which the cumulative mass fractions of particles were 10%, 40%, and 60%, respectively. In addition, measured water content and water pressure head were required for the actual retention curve in order to compare with the result of the FBD model. In this study, the soil water retention data of 30 different soils, measured by Yu et al.^50^, Chen and Wang^51^, Zhang and Miao^52^, Liu and Amemiya^53^, Hayano et al.^54^, and Yabashi et al.^55^ were used for model verification (Table 2). The data covered soils in China (such as black soil, chernozem soil, cinnamon soil, brown earth, fluvo-aquic soil, albic soil, red earth, humid-thermo ferralitic, purplish soil, meadow soil, and yellow earth) and soils in Japan (such as volcanic ash soil and acolian sandy soil). The USDA soil taxonomy of these soils was provided in Table 2. The 30 soils ranged in texture from clay to sand and in bulk density from 0.33 g/cm^3^ to 1.65 g/cm^3^, which covered a much wider range of soil bulk density than many of the existing models or pedotransfer functions^56–59^. Particle size fractions (*D*_*gi*_) were chosen as the upper limit of the diameters between successive sieve sizes. For the data set in which particle density was not determined, 2.65 g/cm^3^ was used.

## Statistical parameters for model verification

Four statistical properties, *R*^2^, *RMSE*, mean residual error (*ME*), and *t* value were calculated to determine the accuracy of the FBD model. The *R*^2^ values were computed at the same value of *ψ*, with the values of *θ* measured and estimated by the FBD model (Eq. 20). *RMSE* and *ME* were obtained, respectively, by

22 $${{RMSE}}\; = \;\left[ {\frac{{1}}{{{n}}}\sum {{(\theta }}_{{{{est}}}} {{ - \theta }}_{{{{mea}}}} {)}^{{2}} } \right]^{{{0}{{.5}}}}$$

23 $${{ME}}\; = \;\frac{{1}}{{{n}}}\sum_{{{i } = { 1}}}^{{{n}}} \left( {{\theta }_{{{{est}}}} - {\theta }_{{{{mea}}}} } \right)$$

where *θ*_*mea*_ was measured soil water content, *θ*_*est*_ was soil water content estimated with the FBD model, and *n* was the number of measured pairs of water content and pressure head. With the assumption of normal distribution and independence of differences between the water contents measured and estimated by the FBD model, *t* was calculated with

24 $${{t}}\; = \;{{ME}}\left( {\frac{{{{RMSE}}^{{2}} - {{ME}}^{{2}} }}{{{{n}} - {1}}}} \right)^{{ - {0}{{.5}}}}$$

when calculated |*t*| was larger than *t*_0.05_ (the critical value of the Student’s *t* distribution for *P* = 0.05 and *n*−1 degrees of freedom), the differences between the measured and estimated water contents were statistically significant. If *t* < 0, soil water contents were underestimated and vice versa. Thus, *t* was a measure for the systematic bias in the estimation. Values of *t* close to zero indicated that the measured and estimated soil water contents were not different systematically from each other or, equivalently, that there was no consistent bias. Values of *t* that differed greatly from zero indicated the presence of systematic bias. *RMSE* was a measure for the scatter of the data points around the 1:1 line. Low *RMSE* values indicated less scatter. Low *RMSE* values also implied low *ME*. Regarding the result that *t* was low while *RMSE* was high, it could be explained that negative and positive deviations distributed more evenly on the two sides of 1:1 line.
